# Assessment of Thermal Influence on an Orthodontic System by Means of the Finite Element Method

**DOI:** 10.3390/bioengineering11101002

**Published:** 2024-10-07

**Authors:** Stelian-Mihai-Sever Petrescu, Anne-Marie Rauten, Mihai Popescu, Mihai Raul Popescu, Dragoș Laurențiu Popa, Dumitru Ilie, Alina Duță, Laurențiu Daniel Răcilă, Daniela Doina Vintilă, Gabriel Buciu

**Affiliations:** 1 Department of Orthodontics, Faculty of Dental Medicine, University of Medicine and Pharmacy of Craiova, 200349 Craiova, Romania; mihaipetrescu2702@gmail.com (S.-M.-S.P.); rautenannemarie@yahoo.com (A.-M.R.); 2 Department of Pedodontics, Faculty of Dental Medicine, University of Medicine and Pharmacy of Craiova, 200349 Craiova, Romania; mihai_15005@yahoo.com; 3 Department of Occlusology and Fixed Prosthetics, Faculty of Dental Medicine, University of Medicine and Pharmacy of Craiova, 200349 Craiova, Romania; 4 Department of Automotive, Transportation and Industrial Engineering, Faculty of Mechanics, University of Craiova, 200478 Craiova, Romania; dumitru_ilie@yahoo.com (D.I.); alina.duta@edu.ucv.ro (A.D.); 5 Department of Applied Mechanics, Faculty of Mechanics, University of Craiova, 200478 Craiova, Romania; racila_laurentiu@yahoo.com (L.D.R.); vintila_dnl@yahoo.com (D.D.V.); 6 Department of General Nursing, Faculty of Nursing-Târgu Jiu, Titu Maiorescu University, 210102 Târgu Jiu, Romania; buciugabriel@yahoo.com

**Keywords:** cone beam computed tomography, dental structures, finite element method, orthodontics, temperature maps

## Abstract

The development of the finite element method (FEM) combined block polynomial interpolation with the concepts of finite difference formats and the variation principle. Because of this combination, the FEM overcomes the shortcomings of traditional variation methods while maintaining the benefits of current variation methods and the flexibility of the finite difference method. As a result, the FEM is an advancement above the traditional variation methods. The purpose of this study is to experimentally highlight the thermal behavior of two stomatognathic systems, one a control and the other presenting orthodontic treatment by means of a fixed metallic orthodontic appliance, both being subjected to several thermal regimes. In order to sustain this experimental research, we examined the case of a female subject, who was diagnosed with Angle class I malocclusion. The patient underwent a bimaxillary CBCT investigation before initiating the orthodontic treatment. A three-dimensional model with fully closed surfaces was obtained by using the InVesalius and Geomagic programs. Like the tissues examined in the patient, bracket and tube-type elements as well as orthodontic wires can be included to the virtual models. Once it is finished and geometrically accurate, the model is exported to an FEM-using program, such as Ansys Workbench. The intention was to study the behavior of two stomatognathic systems (with and without a fixed metallic orthodontic appliance) subjected to very hot food (70 °C) and very cold food (−18 °C). From the analysis of the obtained data, it was concluded that, following the simulations carried out in the presence of the fixed metallic orthodontic appliance, significantly higher temperatures were generated in the dental pulp.

## 1. Introduction

Malocclusions represent abnormal relationships of the upper and lower arches with or without malpositions of the teeth [1]. They are characterized by the impairment of the formation stages of the dento-maxillary apparatus and have an ever-increasing prevalence as a result of environmental changes. Therefore, every etiopathogenic factor has a role in the more intricate modifications to somatic development [2].

CBCT is the most promising diagnostic imaging method that has appeared recently, being able to provide submillimeter resolutions images for diagnosis of superior quality and with shorter scan times [3].

The applicability of this imaging examination in the oro-maxillo-facial region is due to numerous advantages, such as the low cost, the low dose of radiation to which the patient is exposed, and the small size of the tomograph. At the same time, the disadvantages are few in number: low contrast in soft tissues, image noise, and motion artifacts [4].

The development of the finite element method (FEM) combined block polynomial interpolation with the concepts of finite difference formats and the variation principle. Because of this combination, the FEM overcomes the shortcomings of traditional variation methods while maintaining the benefits of current variation methods and the flexibility of the finite difference method. As a result, the FEM is an advancement above the traditional variation methods [5,6].

The activity of three-dimensional modeling is a process; in general, it is iterative and involves several stages, including recognition of needs, definition of the problem, synthesis, and analysis. A certain component or subsystem of an integrative system is conceptualized by the user, subjected to analysis, improved through the analysis procedure, and remodeled. This process is repeated until the three-dimensional model is optimized by the system of constraints imposed by reality. Components and subsystems are synthesized within the global system in a similar way. Another stage is the evaluation, which consists of determining the degree of achievement of the conditions imposed within the specifications established in the defining of the problem phase. In the last stage, the remodeling and geometric optimization is performed [7].

The objective of this study is to experimentally highlight the thermal behavior of two stomatognathic systems, one a control and the other presenting orthodontic treatment by means of a fixed metallic orthodontic appliance, both being subjected to several thermal regimes.

## 2. Materials and Methods

The present study was approved by the Ethics Committee of the University of Medicine and Pharmacy of Craiova, Romania (approval reference no. 127/09.04.2024), in accordance with the ethical guidelines for research with human participants of the University of Medicine and Pharmacy of Craiova, Romania. Written informed consent was obtained from the patient involved in this study.

In order to sustain our study, we examined the case of a female subject, who presented herself in the Orthodontic Clinic of the Faculty of Dental Medicine of the University of Medicine and Pharmacy in Craiova, Romania.

After an orthodontic examination, we found that our subject presented an Angle class I malocclusion, dento-alveolar disharmony with crowding. It was decided to apply a fixed metallic orthodontic appliance, using the straight-wire technique.

At the same time, for this research, the patient underwent a bimaxillary CBCT imaging investigation. A set of 586 tomographic images was used.

A Lenovo laptop computing system with the following technical characteristics was used: INTEL Core I5 processor with a frequency of 2.9 GHz; classic hard disk of 930 GB; RAM memory of 16 Gb; SSD hard disk of 476 GB; Windows 10 64-bit operating system.

The thermal simulations applied to the models with the help of the finite element method, the processing and organization of the obtained data, and the data for the generation of virtual models, were obtained with a Hewlett Packard graphics station with the following main technical characteristics: processor-Intel^®^ Core™ i9-13900K (up to 5.8 GHz with Intel^®^ Turbo Boost Technology, 36 MB L3 cache, 24 cores); processor cache-L3 of 36 MB; family-13th generation Intel^®^ Core™ i9 processor; memoryRAM MHz DDR5-4800 of 64 GB (4 × 16 GB); graphics-NVIDIA RTX™ A4500 (20 GB dedicated GDDR6 memory); graphics-Intel^®^ UHD Graphics; memory and storage-64 GB memory/TB SSD storage; memory Slots-4 DIMMs; internal Storage-SSD HP Z Turbo Drive PCIe^®^ NVMe™ TLC of 1 TB; operating system-Windows 11 Pro.

InVesalius 3.1.1 (CTI, Campinas, Brazil) is a free-to-download program for the generation of three-dimensional geometric structures starting from sets of images obtained by computed tomography (CT or CBCT) or by magnetic resonance. The program is dedicated to research in the medical field which, based on the different shades of gray of the human body tissues, allows the obtaining of a primary geometry. This primary geometry consists of “clouds of points” and it is contained in stereolithography type files (obj, stl), similar to those obtained by three-dimensional scanning.

Geomagic Wrap 2021.2.0 (3D Systems, Rock Hill, SC, USA) is a program dedicated to reverse engineering that allows the processing of files that contain “clouds of points”. It is known that the primary geometry of tissues contains so-called artifacts, i.e., virtual objects that do not exist in the human body, but which appear on tomographic images, due to the effects generated by the refraction and reflection of X-rays. Also, this software allows the elimination of non-conforming surfaces, self-intersections surfaces, etc.

SolidWorks 2021 (Dassault Systèmes, Velizy-Villacoublay, France) is a computer-aided design (CAD) program that uses techniques and methods specific to direct engineering. This software allows the chaining of shapes to generate a virtual solid, but also the operation in environments specific to multi-body models. For example, SolidWorks automatically transforms a completely closed surface created in Geomagic into a virtual solid. Ansys Workbench 19.2 (Ansys, Inc., Canonsburg, PA, USA) is a software that allows the simulation of a multitude of physical phenomena applied to some virtual solid models and allows obtaining their physical behavior based on the result maps. This program operates with algorithms specific to the finite element method.

This program package (Microsoft Corporation, Redmond, WA, USA) was used to systematize and interpret the data obtained from the result maps given by the simulations in Ansys, but also to obtain some figures, graphs, or diagrams.

For the development of thermal simulations, the following techniques were applied:

- Direct engineering methods, which are included, in particular, in the SolidWorks program, which allow the generation of virtual objects similar to reality models, such as orthodontic wires or brackets and tube-type elements;
- Reverse Engineering methods, which are the basis of the Geomagic program, used for editing and preparing models that, initially, were composed of the so-called “clouds of points”;
- Thermodynamics methods that were used to define the simulations made in Ansys Workbench;
- Techniques specific to the finite element method that are the basis of the algorithms contained in the thermal modules of the Ansys program.

### 2.1. Elaboration of the Three-Dimensional Model of a Stomatognathic System Quasi-Identical to the Patient’s

**Figure 1:**
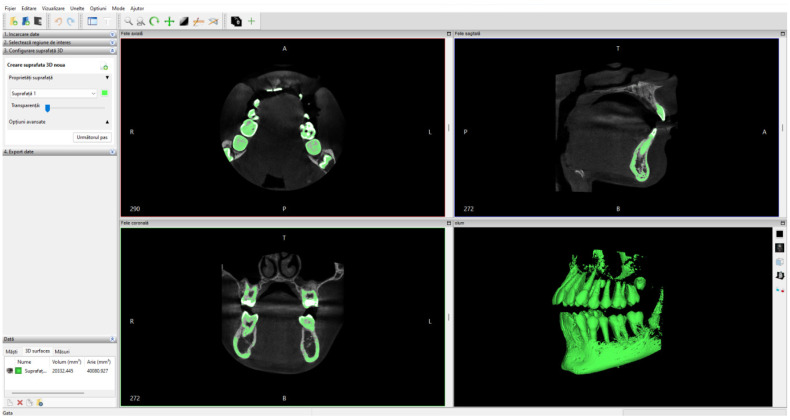
The activation of the Enamel filter in the InVesalius program.

Initially, the set of CBCT tomographic images was loaded into the InVesalius program and the Enamel filter was used for the dental enamel. Figure 1 shows the interface of this program.

**Figure 2:**
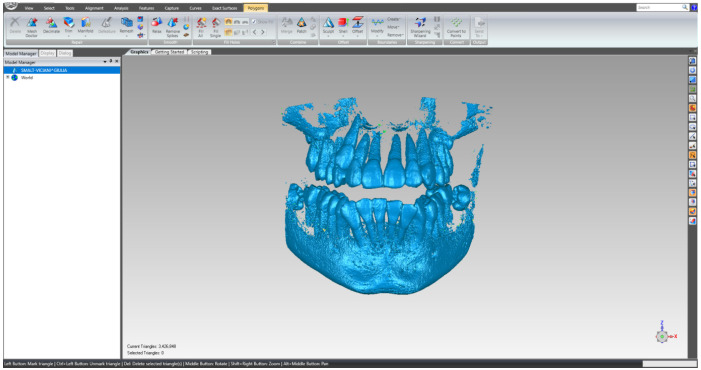
The primary geometry loaded in the Geomagic program.

This primary “cloud of points” geometry was loaded in Geomagic in order to be edited and processed, as can be seen in Figure 2.

**Figure 3:**
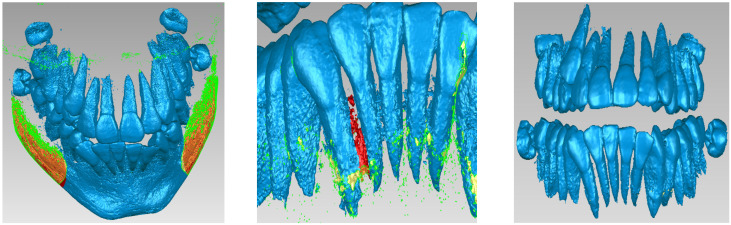
Steps to remove the bone components from the initial geometry in Geomagic.

Due to the similar density of the mandible and maxilla bones compared to the density of dental enamel, the areas of these bones were found in this initial model. For this reason, these areas were removed manually. The stages of these removal operations are shown in Figure 3.

**Figure 4:**
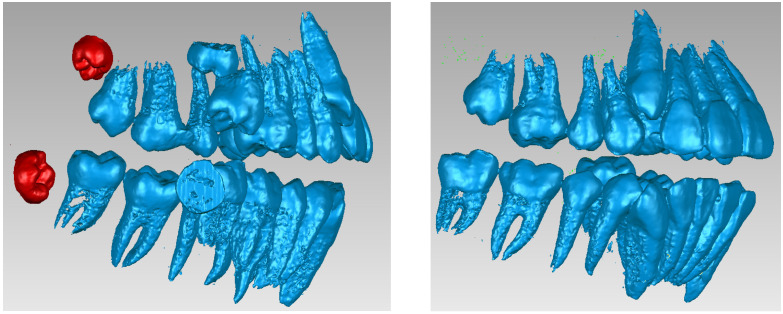
Removing the wisdom teeth from the dentition model.

Next, the wisdom teeth were removed, as can be seen in Figure 4.

**Figure 5:**
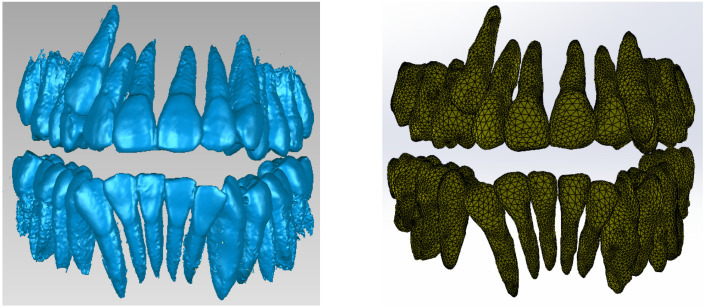
The final model of the dentition in Geomagic and SolidWorks.

Next, the model was subjected to operations to eliminate non-conforming surfaces, but also to other techniques to reduce the number of surfaces, etc. Figure 5 shows the final model of the dentition in Geomagic and also in SolidWorks.

**Figure 6:**
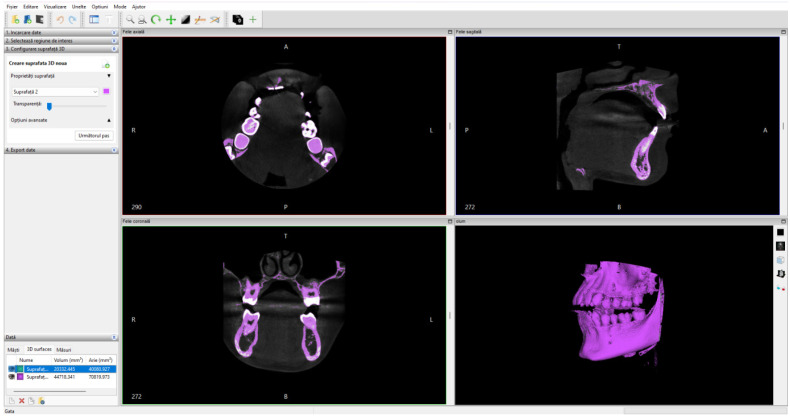
The interface of the InVesalius program with the Compact Bone filter activated.

To obtain the three-dimensional model of the bone components, the CBCT tomographic image set was loaded into the InVesalius program and the Compact Bone filter was activated, as can be seen in Figure 6.

**Figure 7:**
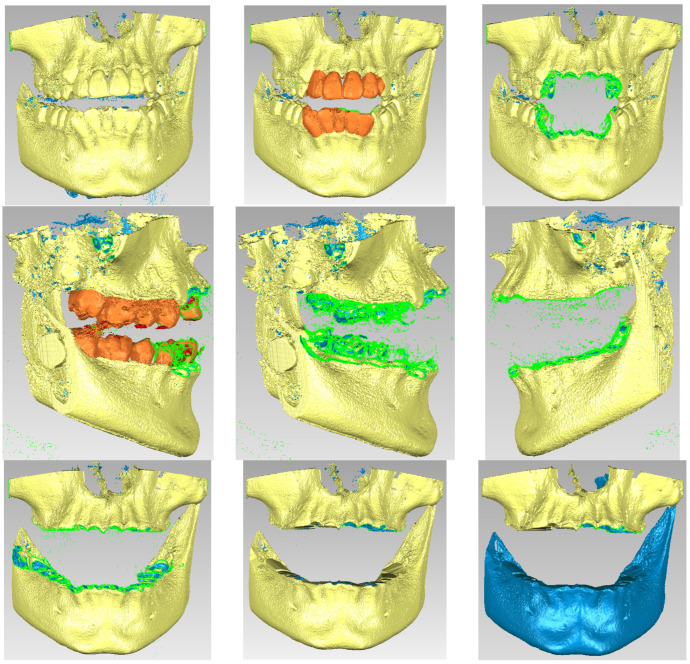
Processing stages of the model in Geomagic.

Due to the fact that bone density is relatively similar to tooth enamel density, it was necessary to remove these dental structures. Figure 7 shows these removal operations.

**Figure 8:**
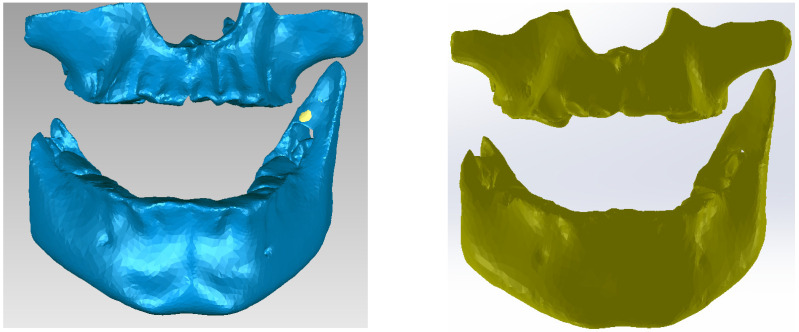
The virtual model of the bone components in Geomagic and in SolidWorks.

Next, specific reverse engineering techniques were applied and the final model of the bone components was obtained. Figure 8 shows the final model in Geomagic and SolidWorks.

**Figure 9:**
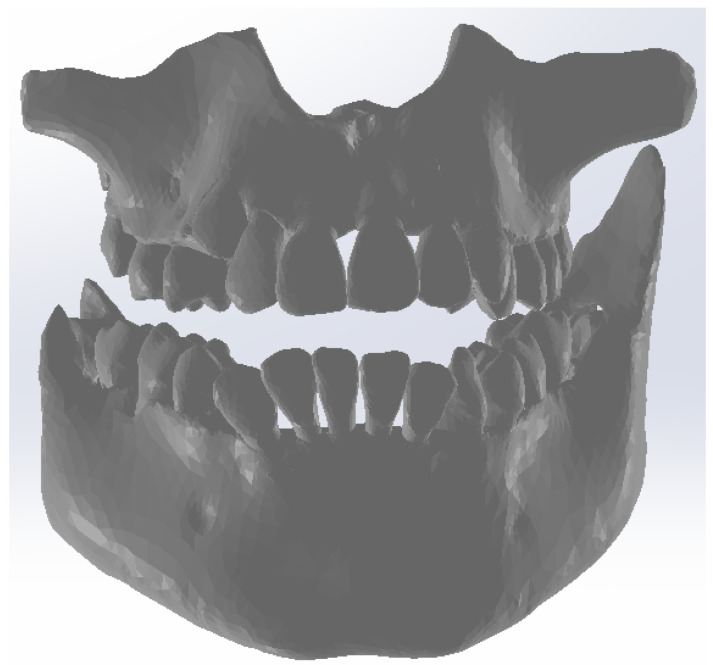
The virtual model of the studied stomatognathic apparatus.

Using known direct engineering techniques and methods, the model of the patient’s stomatognathic apparatus was obtained, as can be seen in Figure 9. When aligning the models, the unique coordinate system was used, which was the same, because the models came from the same CBCT set.

**Figure 10:**
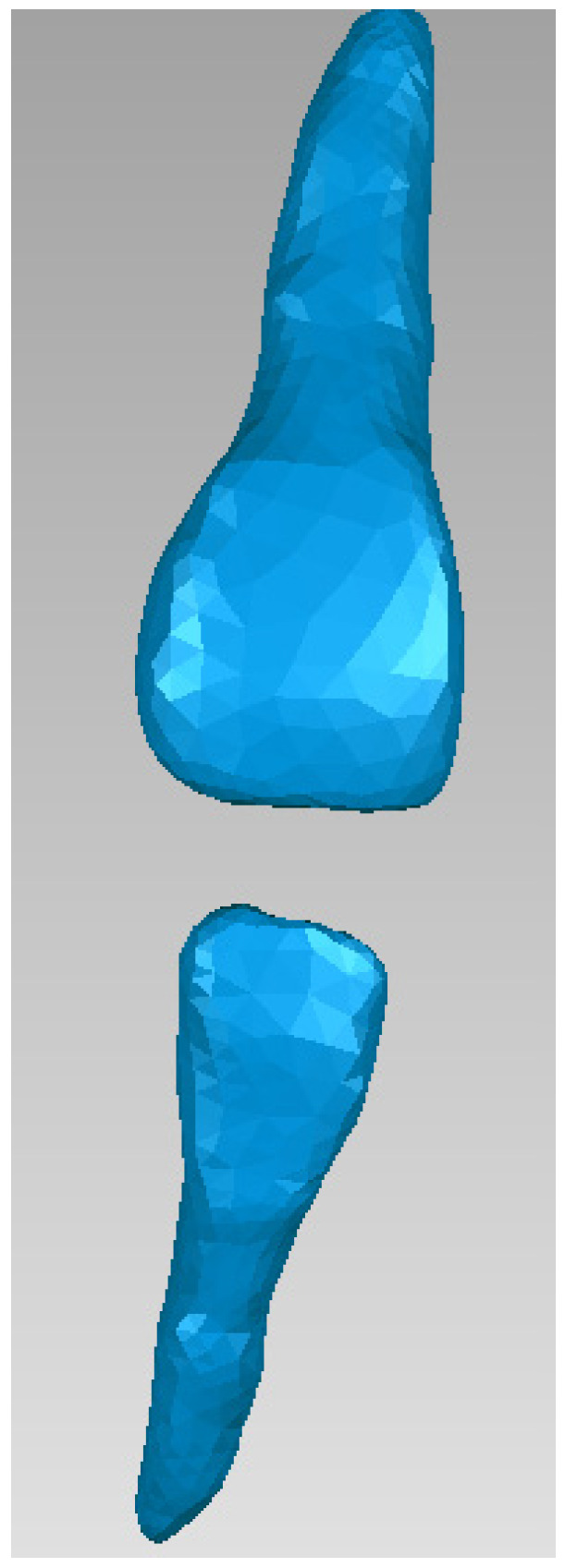
Dental enamel models of teeth 1.1 and 4.1.

To obtain the internal structures of teeth 1.1 and 4.1, initially, the dental enamel models for the two teeth were isolated in Geomagic, as can be seen in Figure 10.

**Figure 11:**
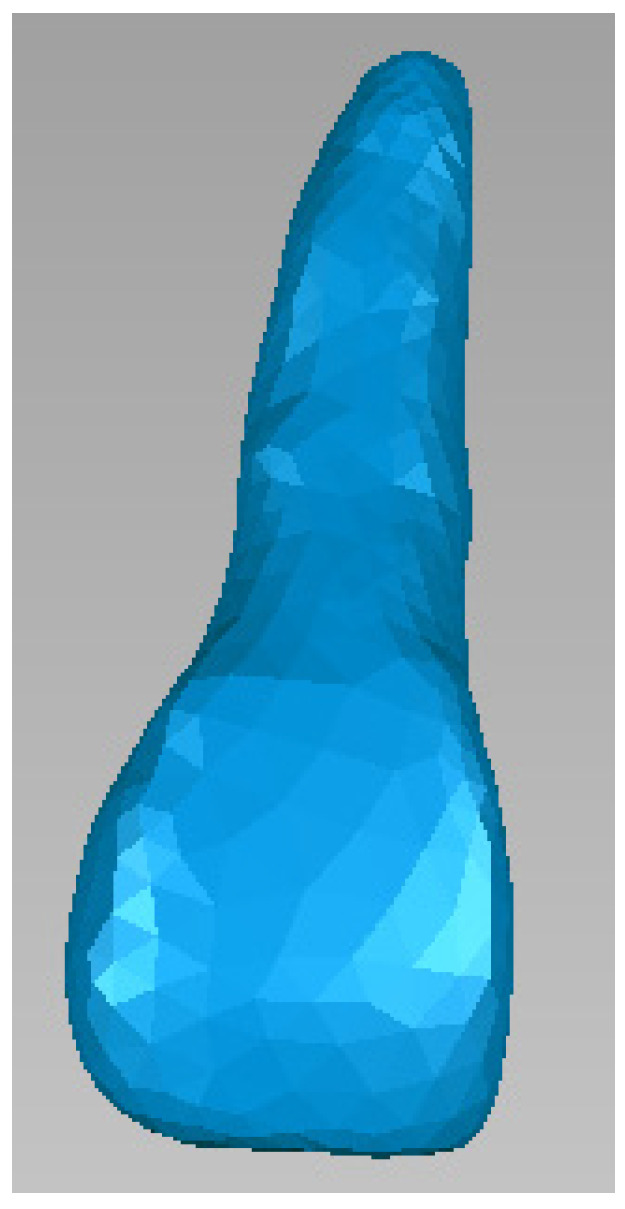
The external model of tooth 1.1 in Geomagic.

Next, tooth 1.1 was isolated, as can be seen in Figure 11.

**Figure 12:**
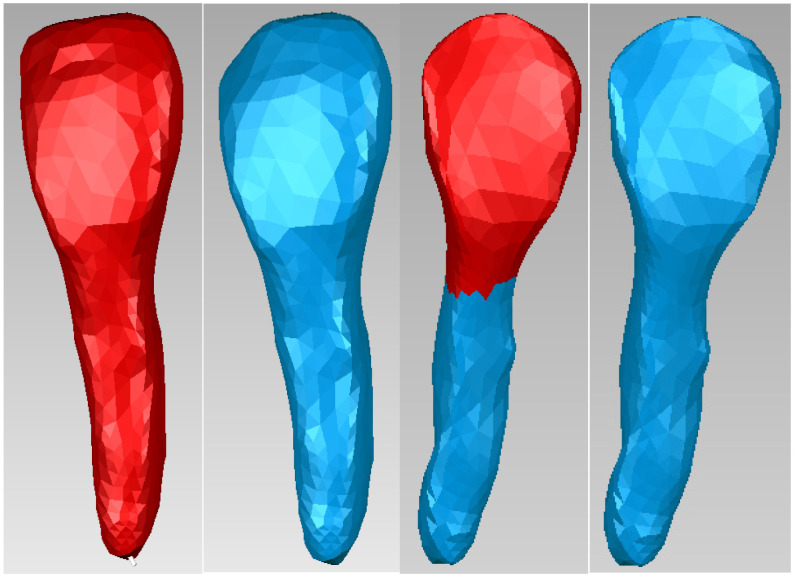
Obtaining the dentine model of tooth 1.1.

Then, offset techniques were applied on the entire model or on some areas of the model to obtain the outer model of the dentin. These operations are shown in Figure 12.

**Figure 13:**
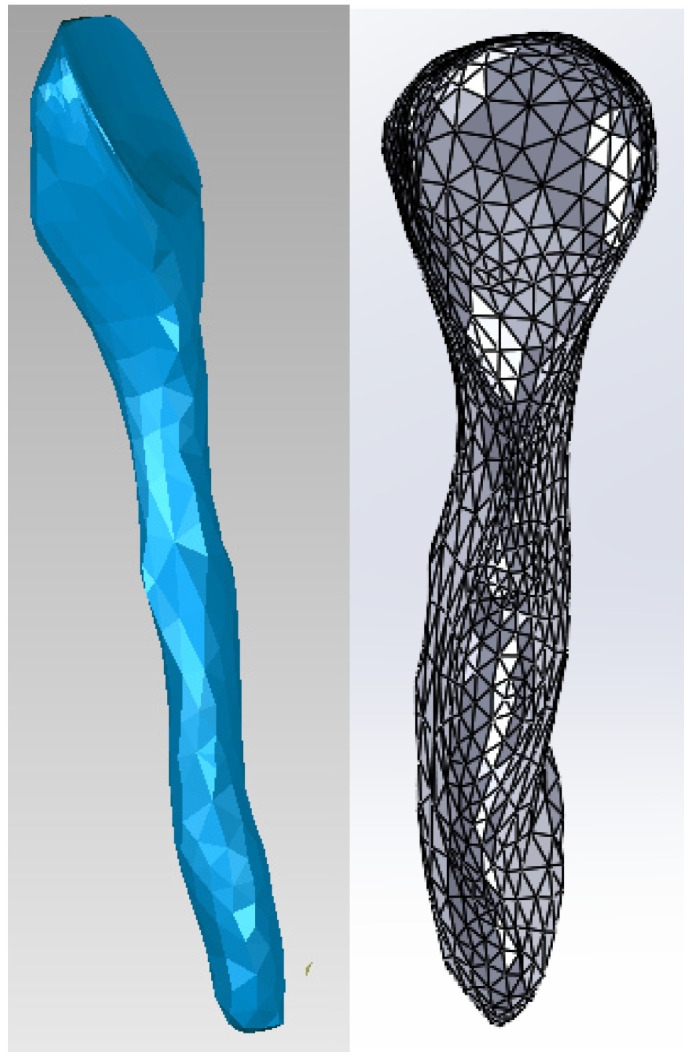
Model of the dental pulp of tooth 1.1 in Geomagic and SolidWorks.

Starting from the dentine model, applying similar offset techniques, the model of tooth 1.1 pulp was finally obtained. Figure 13 shows the final model of tooth 1.1 pulp in Geomagic and SolidWorks.

**Figure 14:**
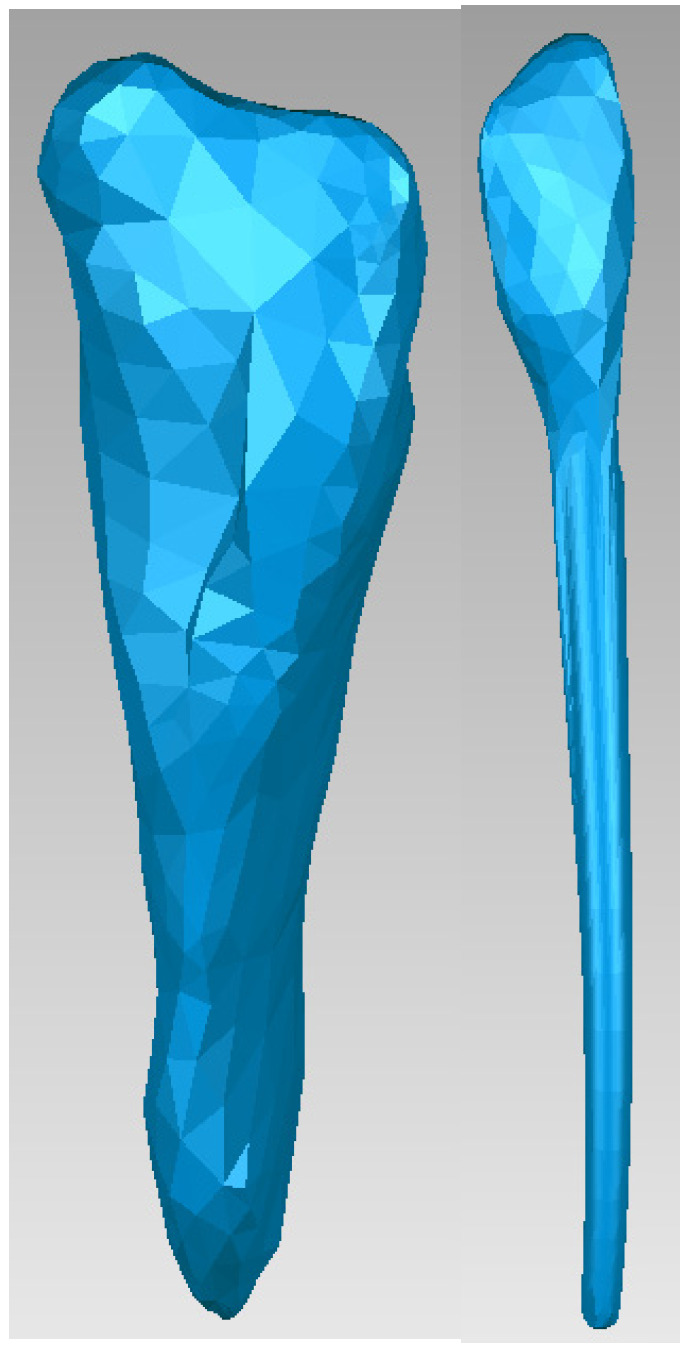
The dentin and dental pulp models of tooth 4.1.

The same procedure was used to obtain the dentine and pulp models for tooth 4.1. Figure 14 shows these models.

**Figure 15:**
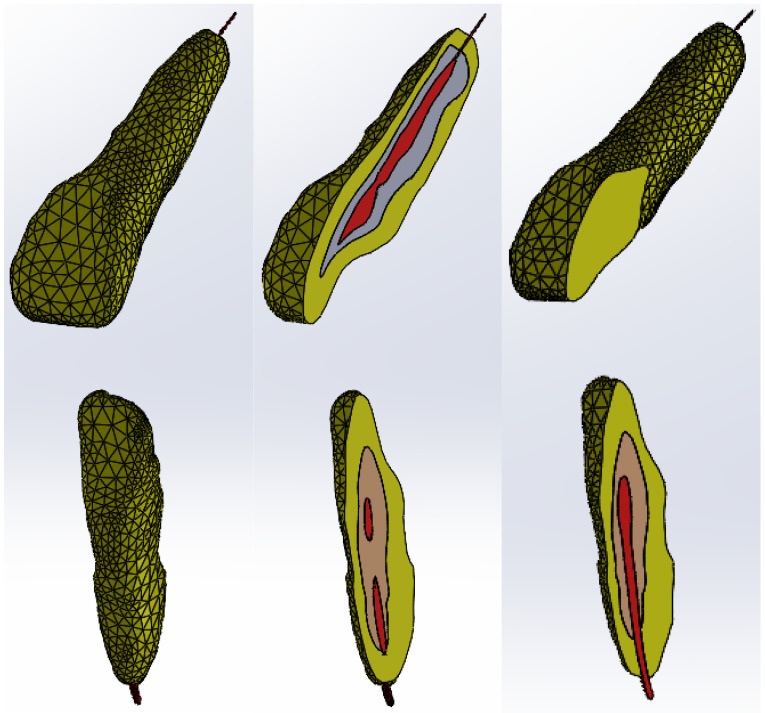
Final models of teeth 1.1 and 4.1 (one view and two sections).

These models of the enamel, dentin, and pulp of teeth 1.1 and 4.1 were loaded into the Assembly module contained in SolidWorks and they were aligned using the coordinate systems. Using volumetric reduction techniques, the final models of teeth 1.1 and 4.1 were obtained, as shown in Figure 15.

**Figure 16:**
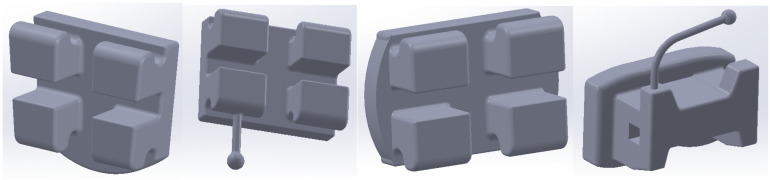
Bracket and tube-type elements.

Using direct engineering techniques and methods, the bracket and tube-type elements were modeled. Figure 16 shows four of these components.

**Figure 17:**
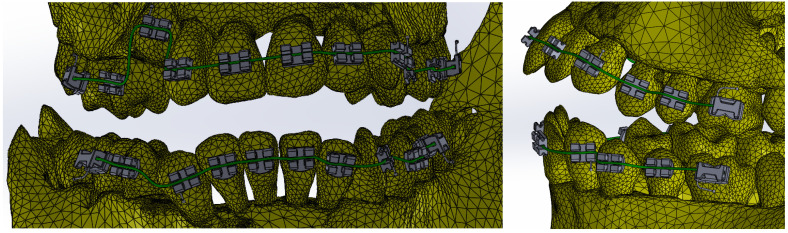
Placement of virtual orthodontic wires.

These components were placed on the teeth according to orthodontic protocols. On each of these components, four points were defined that would be found on the curves defining the orthodontic wires. Initially, two curves were drawn that “connected” these points that were placed above the bracket and tube-type elements, in close proximity. Later, using circular sections placed perpendicular to the curves, sweep-type shapes were defined for the orthodontic wires. Figure 17 shows these orthodontic wires placed on the dentition.

**Figure 18:**
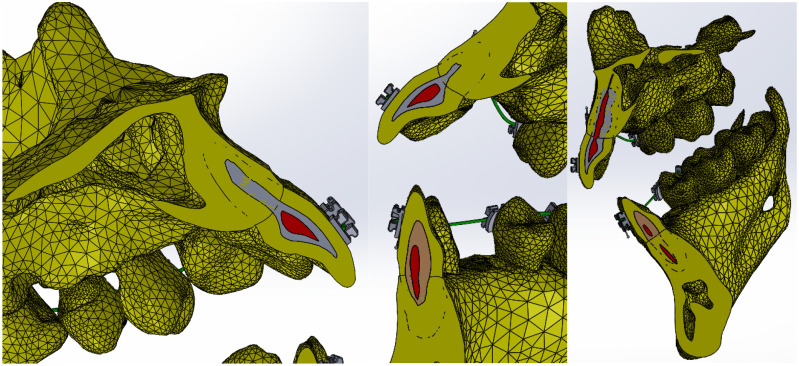
Sections through the studied orthodontic system.

In Figure 18, sections through teeth 1.1 and 4.1 are shown.

### 2.2. Preparation of Thermal Simulations

Two groups of simulations were carried out for the following:

- The model of the control stomatognathic apparatus (in the obtained model, the bracket and tube-type elements and the orthodontic wires were suppressed);
- The model of the stomatognathic apparatus with a fixed metallic orthodontic appliance.

**Figure 19:**
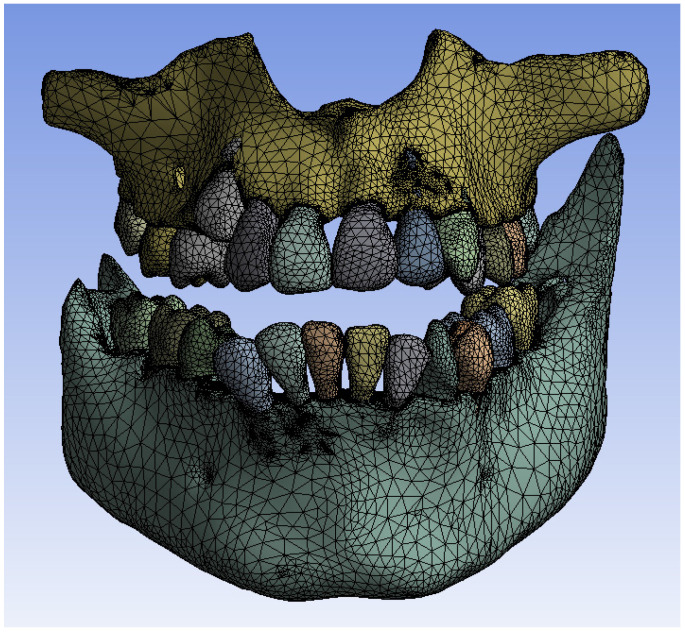
Finite element structure for simulations without a fixed metallic appliance.

These models were loaded into the Transient Thermal module of Ansys Workbench, where they were divided into finite elements. Figure 19 shows the finite element structure composed of 1,248,243 nodes and 726,343 elements for the model without a fixed metallic orthodontic appliance.

**Figure 20:**
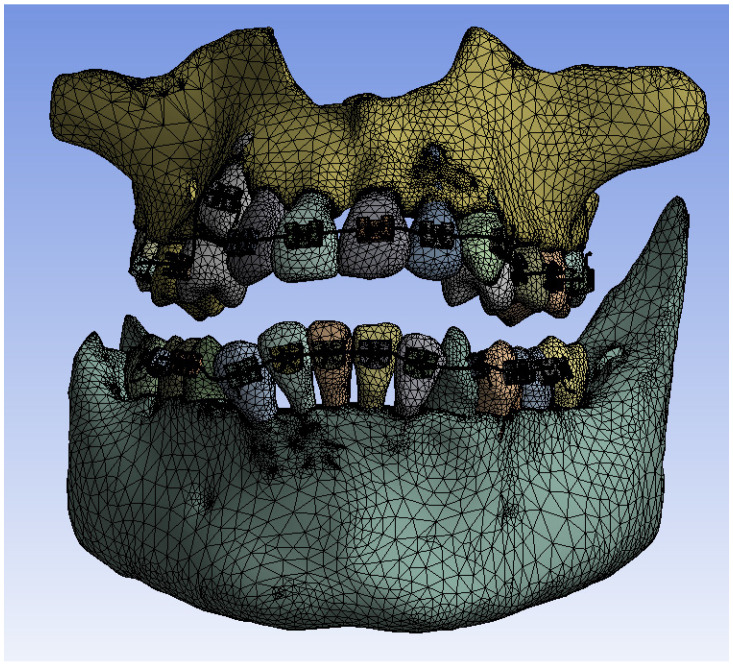
Finite element structure for simulations with a fixed metallic orthodontic appliance.

Figure 20 shows the finite element structure composed of 1,489,889 nodes and 827,771 elements for the model with a fixed metallic orthodontic appliance.

The materials used in the simulations were defined in the Engineering Data module according to their physical and thermal properties. Table 1 shows the materials that were used. Obviously, for the simulations without a fixed metallic orthodontic appliance, the two alloys (Ni + Cr, Ni + Ti) were not used [8,9,10,11,12,13,14,15,16].

**Figure 21:**
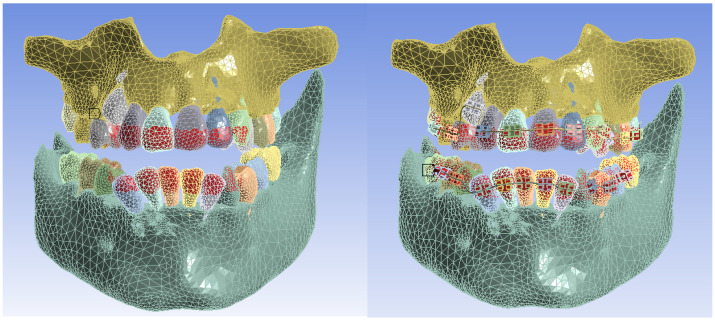
The areas in the models that came into contact with hot or cold food (temperature source).

Next, a temperature source was defined that would materialize by the surfaces and come into contact with hot or cold food, as shown in Figure 21.

**Figure 22:**
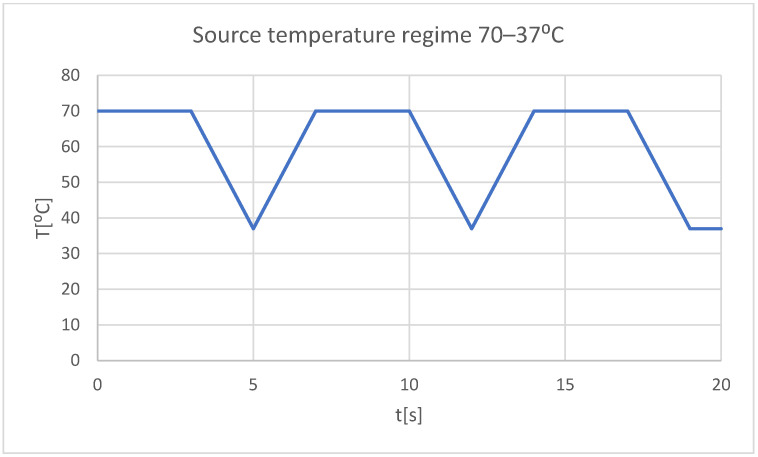
Mode of the heat source (hot food).

**Figure 23:**
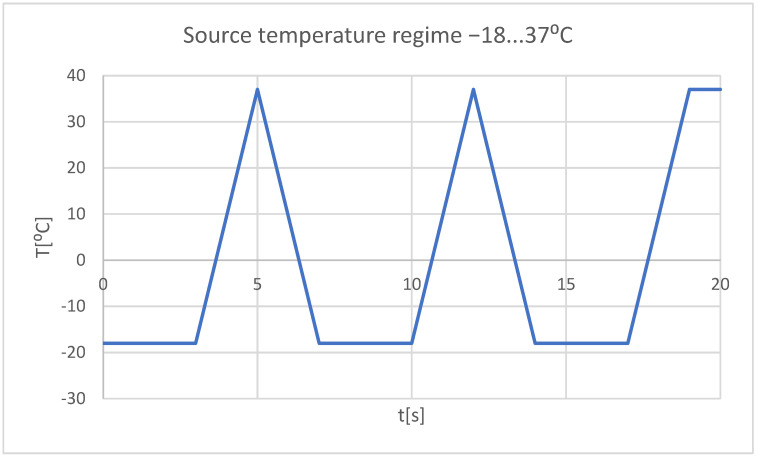
Heat source mode (cold food).

**Figure 24:**
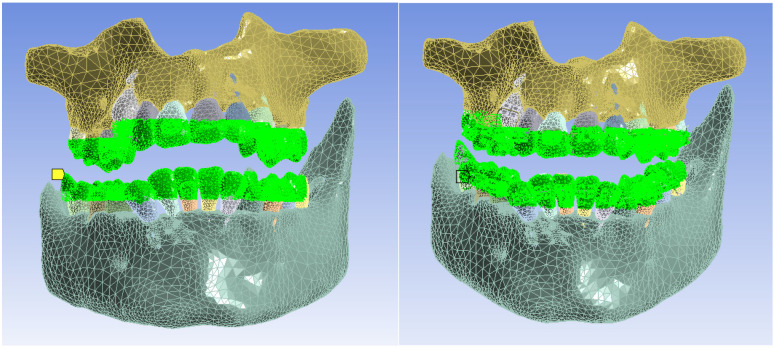
The areas where the phenomenon of thermal convection was present.

We intended to study the behavior of these two systems in different situations, and in particular the following two situations:

- Very hot food (70 °C) that acts for 3 s, then the temperature tends to 37 °C, and the cycle is repeated two more times; the whole regime lasts 20 s (Figure 22);

- Very cold food (−18 °C) that acts for 3 s, then the temperature tends to 37 °C, and the cycle is repeated two more times; the whole regime lasts 20 s (Figure 23);

Next, the area where the convection phenomenon acts was defined, as can be seen in Figure 24. The value of convection in the oral cavity was in the range of 2–3 W/m^2^ [17].

In order to determine the dynamic temperature on the structures of teeth 1.1 and 4.1, some virtual temperature sensors were placed on them (using the Probe command). They recorded the temperature vs. time, the values being taken in a Microsoft Excel type file.

## 3. Results

### 3.1. Thermal Simulation Results for the Stomatognathic Control System with a Hot Temperature Source

**Figure 25:**
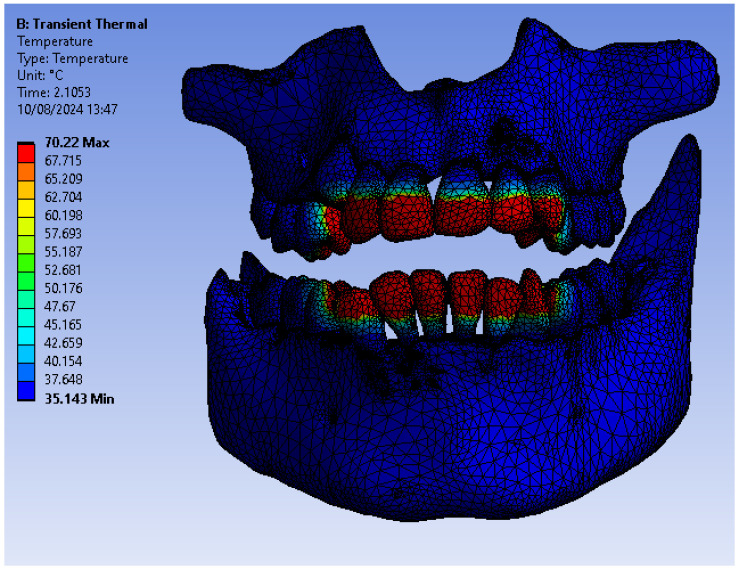
Temperature map.

Figure 25 shows the temperature map of the system subjected to thermal simulation.

**Figure 26:**
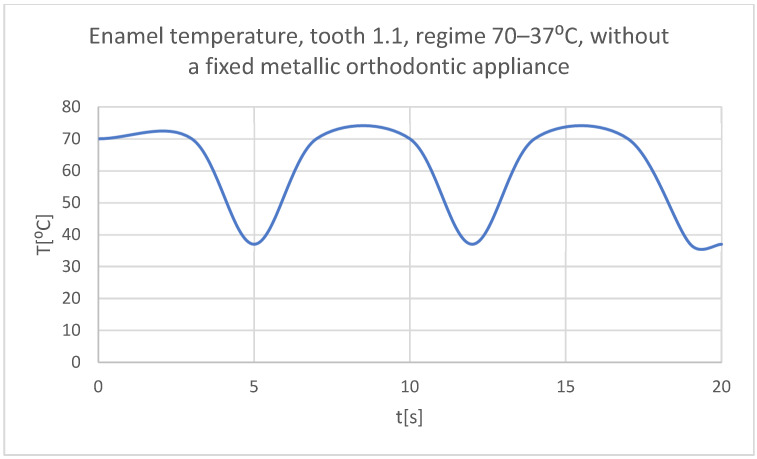
The temperature in the dental enamel of tooth 1.1 subjected to the hot thermal source.

**Figure 27:**
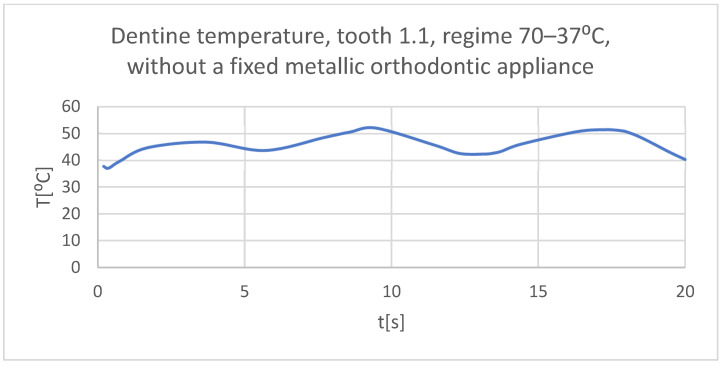
The temperature in the dentine of tooth 1.1 subjected to the hot thermal source.

**Figure 28:**
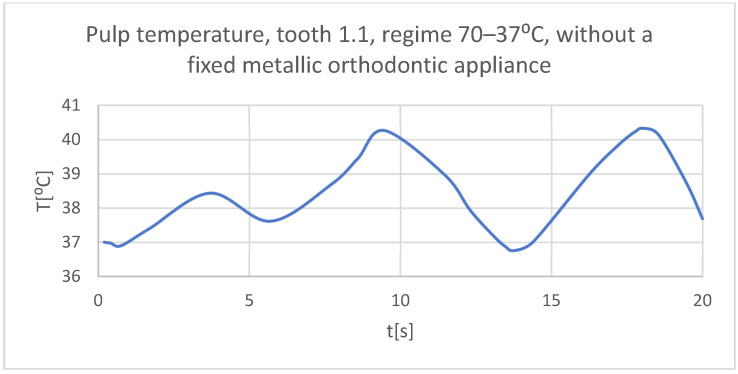
The temperature in the pulp of tooth 1.1 subjected to the hot thermal source.

**Figure 29:**
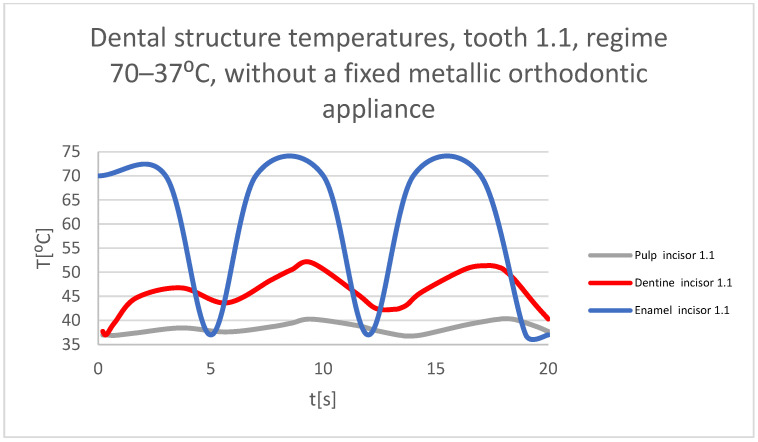
Comparative diagram of the dental structure temperatures of tooth 1.1 subjected to the hot thermal source.

**Figure 30:**
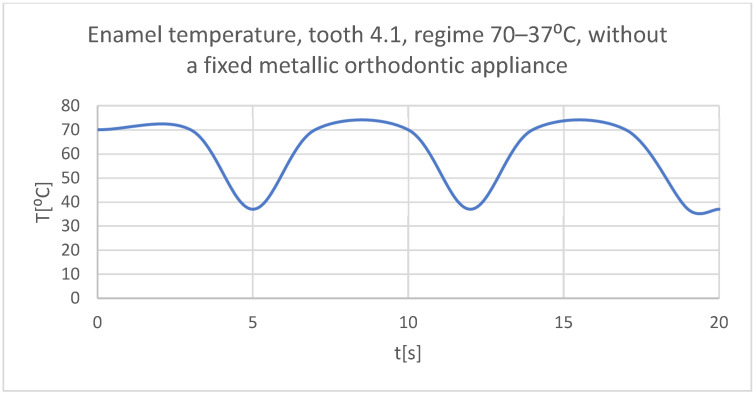
The temperature in the dental enamel of tooth 4.1 subjected to the hot thermal source.

**Figure 31:**
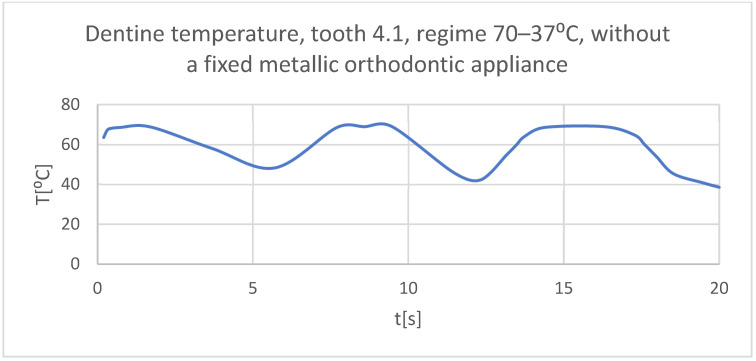
The temperature in the dentin of tooth 4.1 subjected to the hot thermal source.

**Figure 32:**
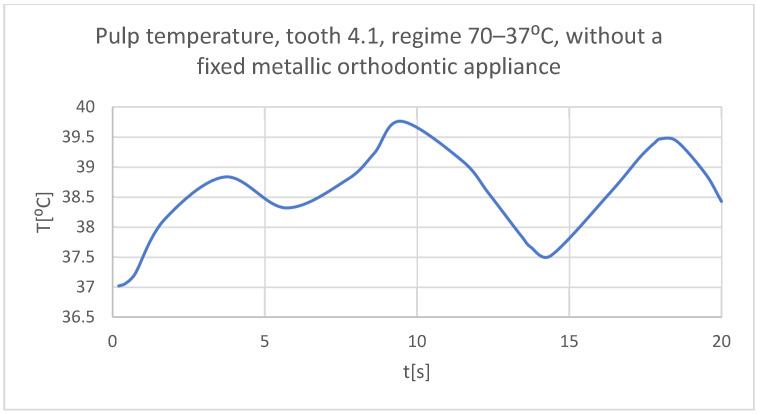
The temperature in the pulp of tooth 4.1 subjected to the hot thermal source.

**Figure 33:**
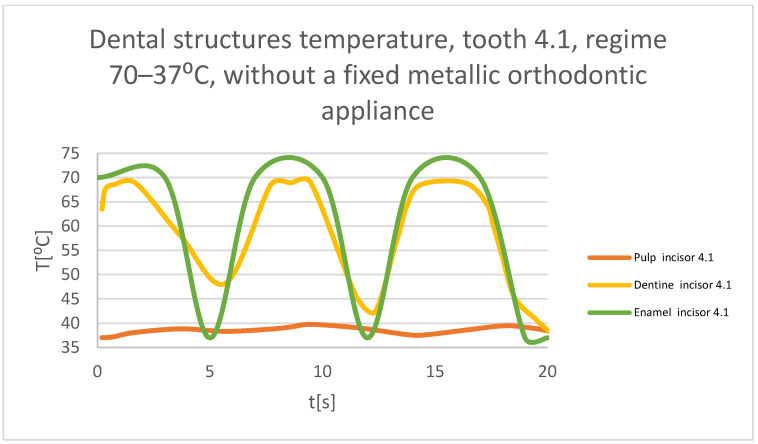
Comparative diagram of the dental structures temperatures of tooth 4.1 subjected to the hot thermal source.

**Figure 34:**
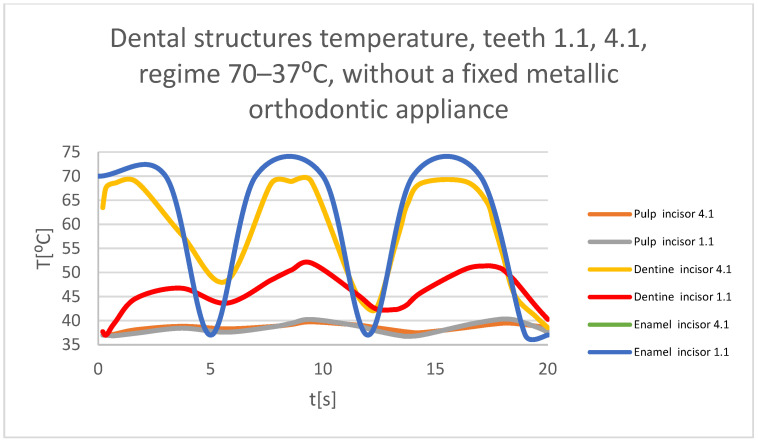
Comparative diagram of the dental structure temperatures of teeth 1.1 and 4.1 subjected to the hot thermal source.

Using the Probe-type virtual temperature sensors, the temperature diagrams of the dental enamel (Figure 26), of the dentine (Figure 27), and of the pulp (Figure 28) were obtained for tooth 1.1, the comparative diagram of the dental structures was obtained for tooth 1.1 (Figure 29), the temperature diagrams of the dental enamel (Figure 30), of the dentine (Figure 31), of the pulp (Figure 32) were obtained for tooth 4.1, the comparative diagram of the dental structures was obtained for tooth 4.1 (Figure 33), and the comparative diagram of the dental structures was obtained for teeth 1.1 and 4.1 (Figure 34).

### 3.2. Thermal Simulation Results for the Stomatognathic Control System with a Cold Temperature Source

**Figure 35:**
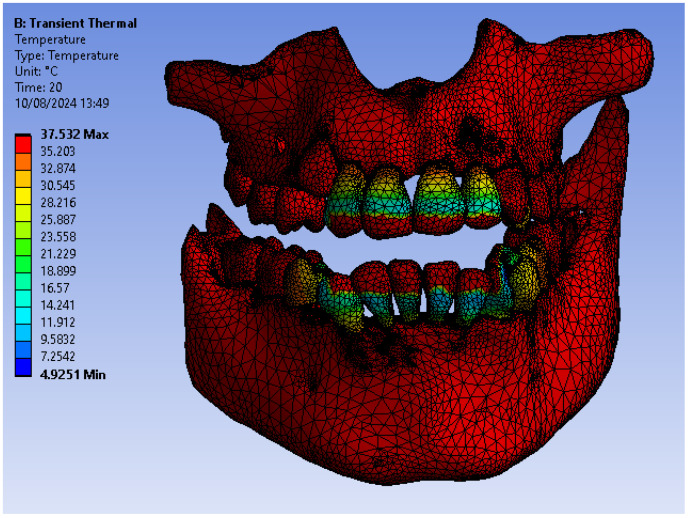
Temperature map.

Figure 35 shows the temperature map of the system subjected to thermal simulation.

**Figure 36:**
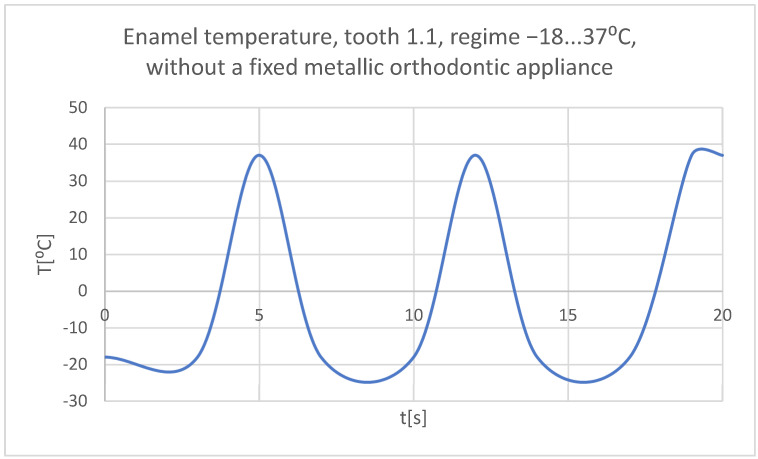
The temperature in the dental enamel of tooth 1.1 subjected to the cold thermal source.

**Figure 37:**
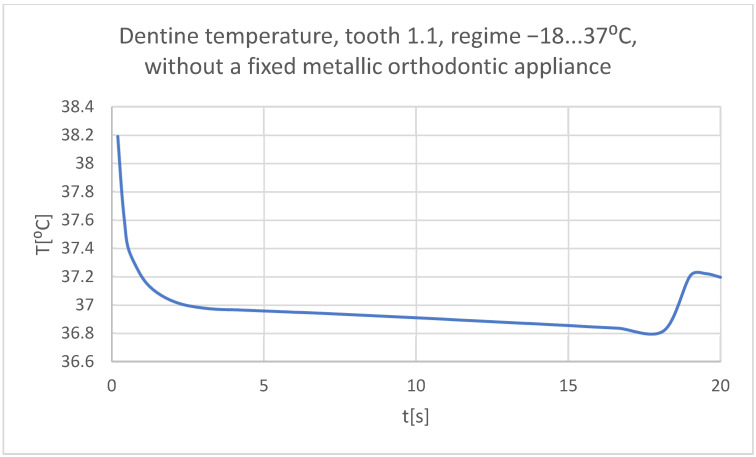
The temperature in the dentine of tooth 1.1 subjected to the cold thermal source.

**Figure 38:**
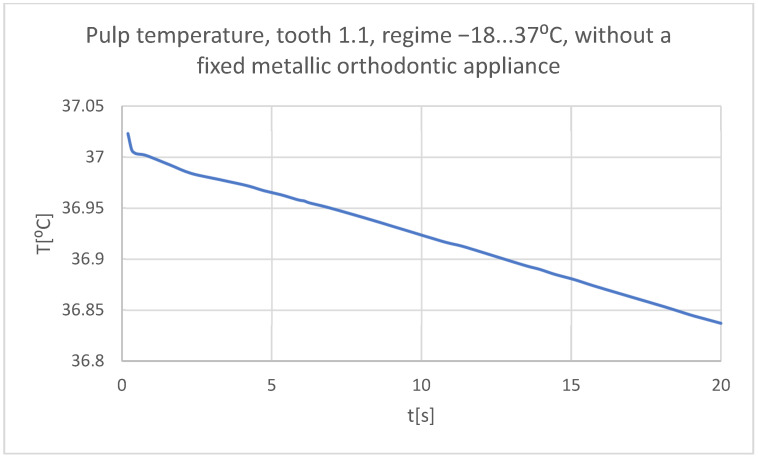
The temperature in the pulp of tooth 1.1 subjected to the cold thermal source.

**Figure 39:**
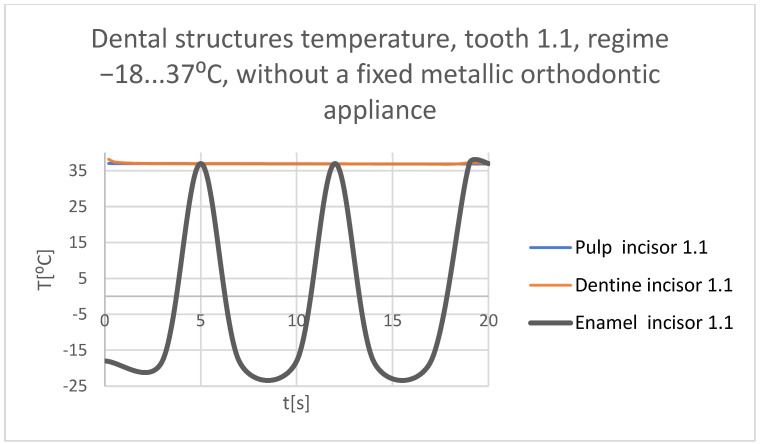
Comparative diagram of the dental structures temperatures of tooth 1.1 subjected to the cold thermal source.

**Figure 40:**
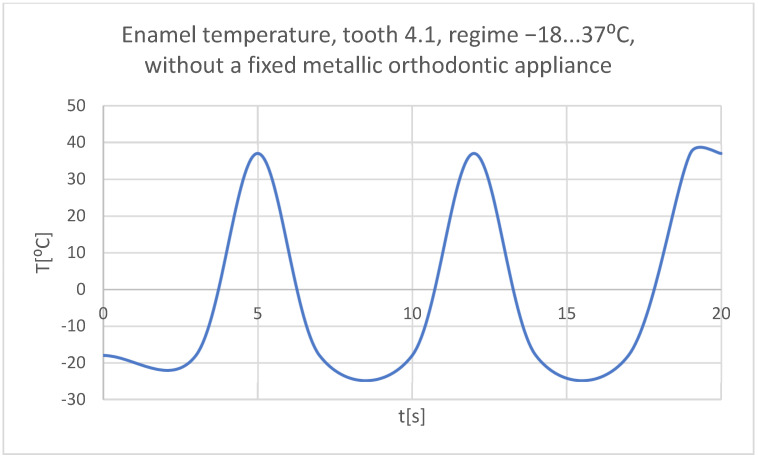
The temperature in the dental enamel of tooth 4.1 subjected to the cold thermal source.

**Figure 41:**
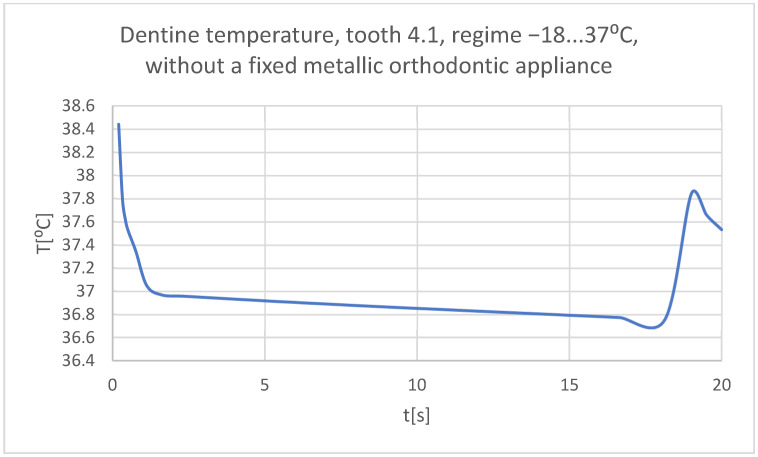
The temperature in the dentin of tooth 4.1 subjected to the cold thermal source.

**Figure 42:**
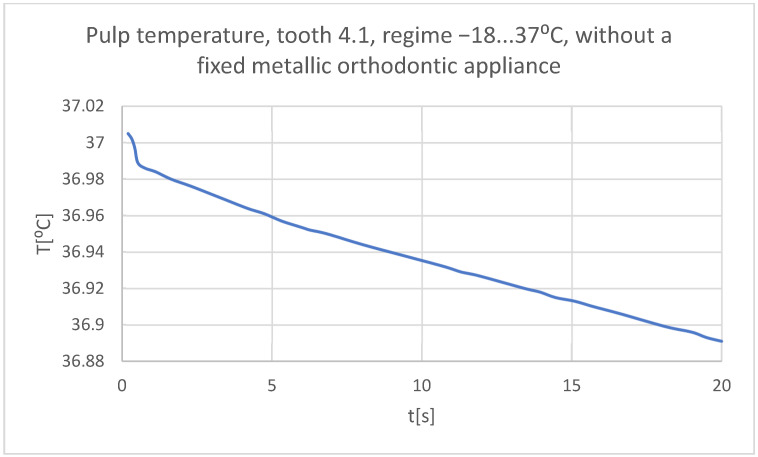
The temperature in the pulp of tooth 4.1 subjected to the cold thermal source.

**Figure 43:**
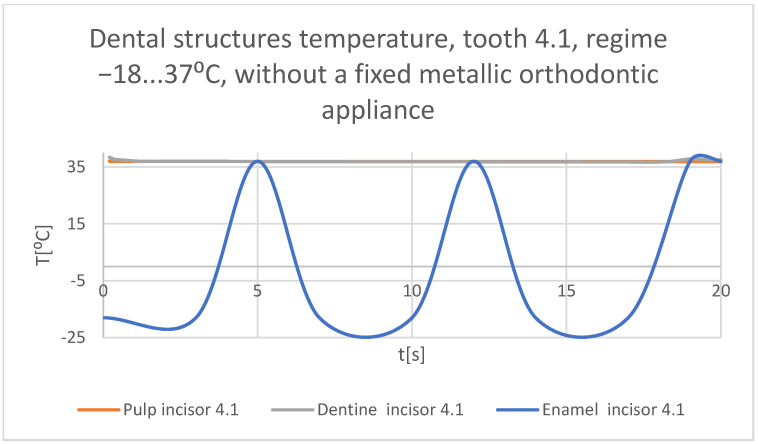
Comparative diagram of the dental structure temperatures of tooth 4.1 subjected to the cold thermal source.

**Figure 44:**
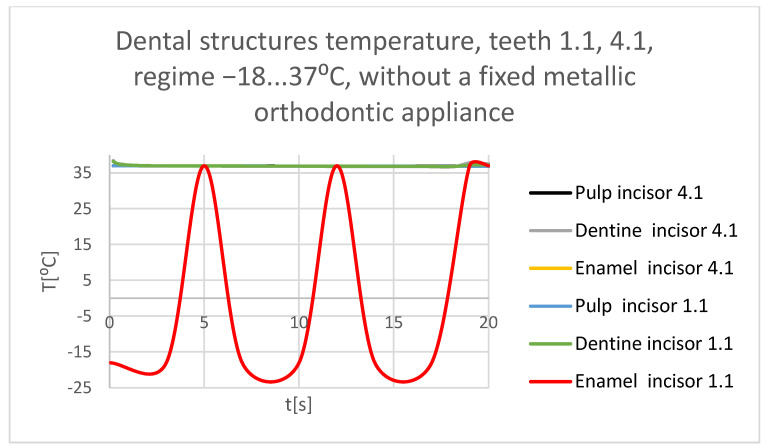
Comparative diagram of the dental structure temperatures of teeth 1.1, 4.1 subjected to the cold thermal source.

Using the Probe-type virtual temperature sensors, the temperature diagrams of the tooth enamel (Figure 36), of the dentine (Figure 37), and of the pulp (Figure 38) were obtained for tooth 1.1, the comparative diagram of the dental structures was obtained for tooth 1.1 (Figure 39), the temperature diagrams of the dental enamel (Figure 40), of the dentine (Figure 41), and of the pulp (Figure 42) were obtained for tooth 4.1, the comparative diagram of the dental structures was obtained for tooth 4.1 (Figure 43), and the comparative diagram of the dental structures was obtained for teeth 1.1 and 4.1 (Figure 44).

### 3.3. The Results of the Thermal Simulation for the Stomatognathic System with a Fixed Metallic Orthodontic Appliance Having a Hot Temperature Source

**Figure 45:**
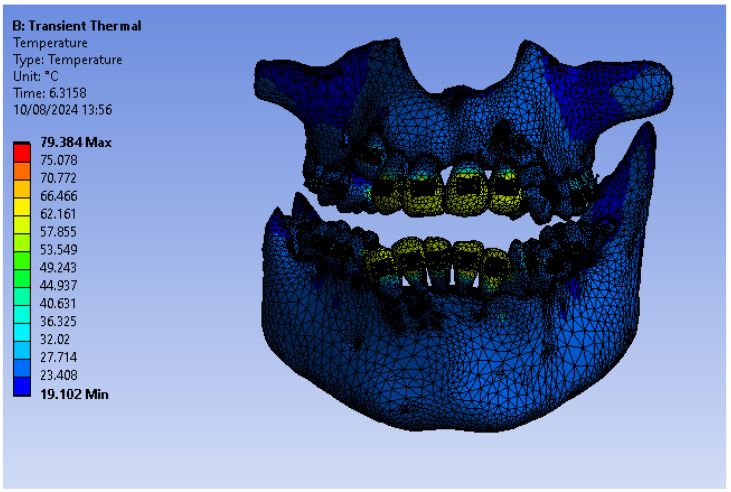
Temperature map.

Figure 45 shows the temperature map of the system subjected to thermal simulation.

**Figure 46:**
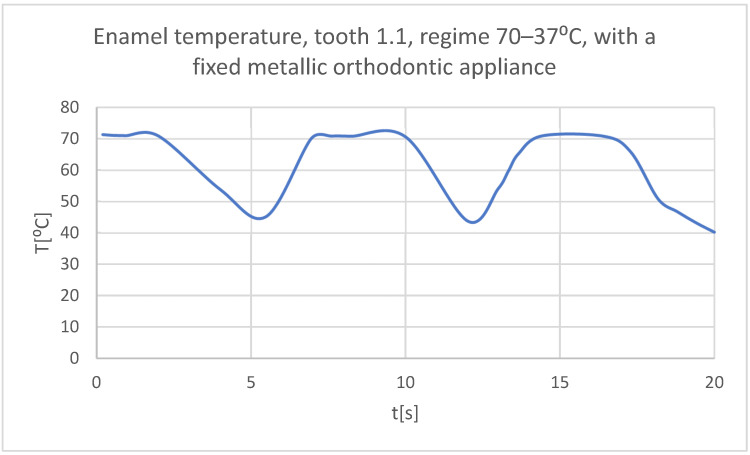
The temperature in the dental enamel of tooth 1.1 subjected to the hot thermal source.

**Figure 47:**
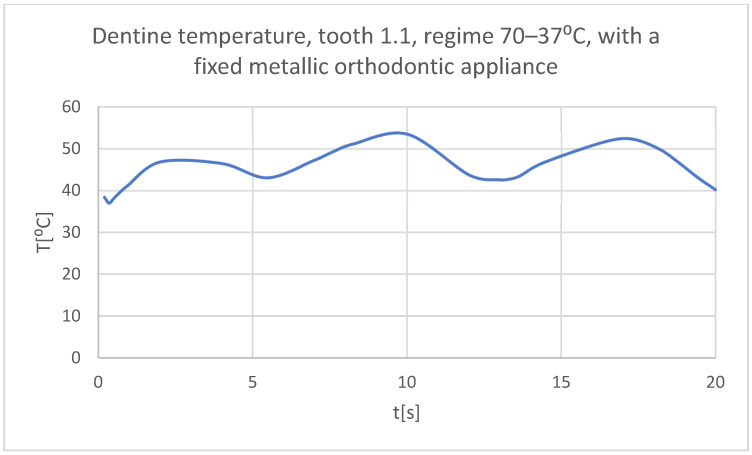
The temperature in the dentine of tooth 1.1 subjected to the hot thermal source.

**Figure 48:**
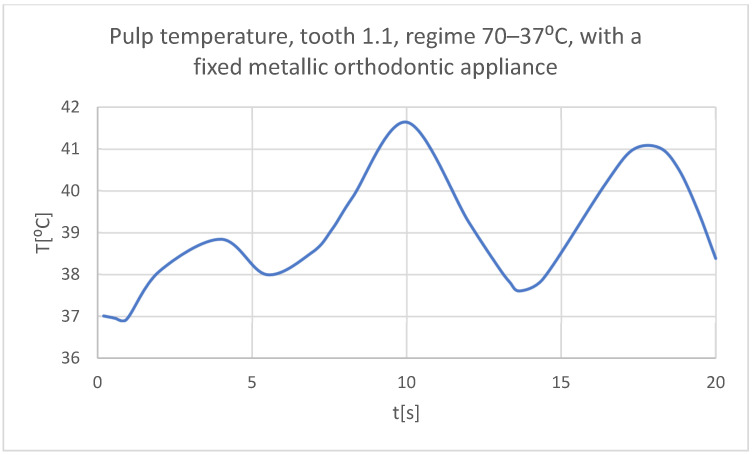
The temperature in the pulp of tooth 1.1 subjected to the hot thermal source.

**Figure 49:**
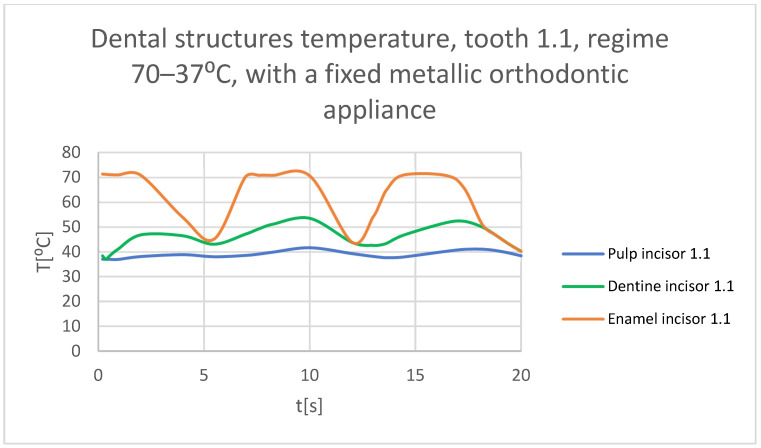
Comparative diagram of the dental structures temperature of tooth 1.1 subjected to the hot thermal source.

**Figure 50:**
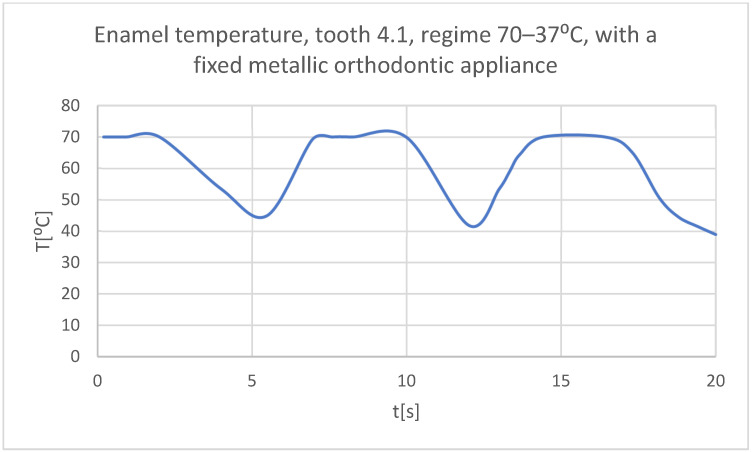
The temperature in the dental enamel of tooth 4.1 subjected to the hot thermal source.

**Figure 51:**
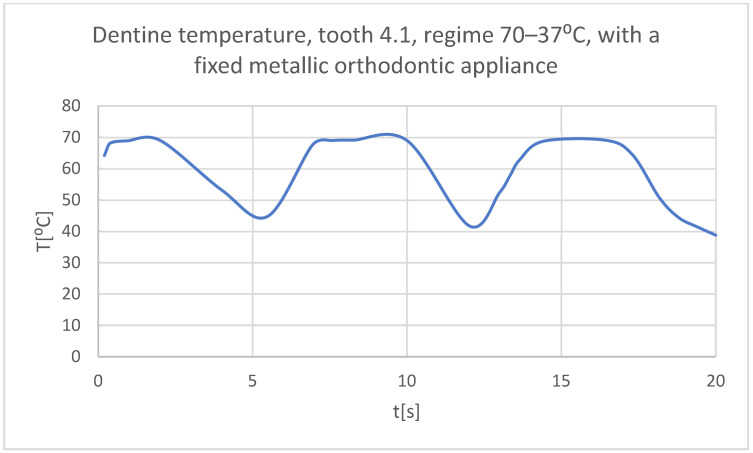
The temperature in the dentin of tooth 4.1 subjected to the hot thermal source.

**Figure 52:**
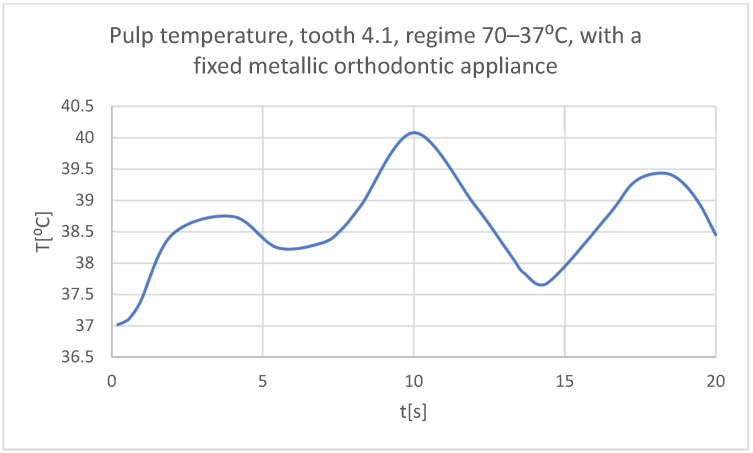
The temperature in the pulp of tooth 4.1 subjected to the hot thermal source.

**Figure 53:**
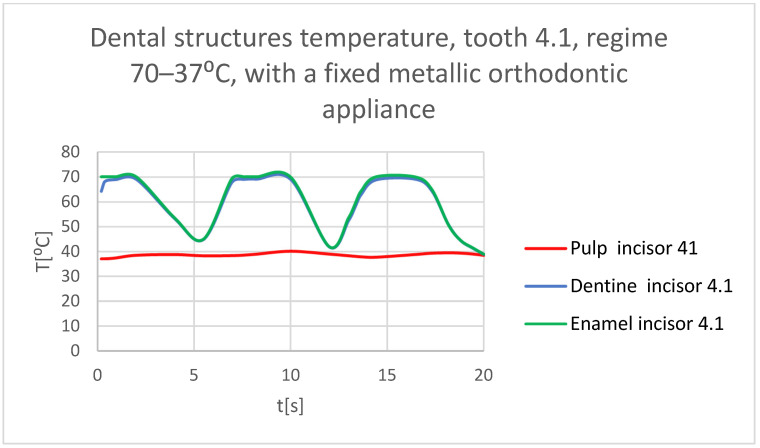
Comparative diagram of the dental structure temperature of tooth 4.1 subjected to the hot thermal source.

**Figure 54:**
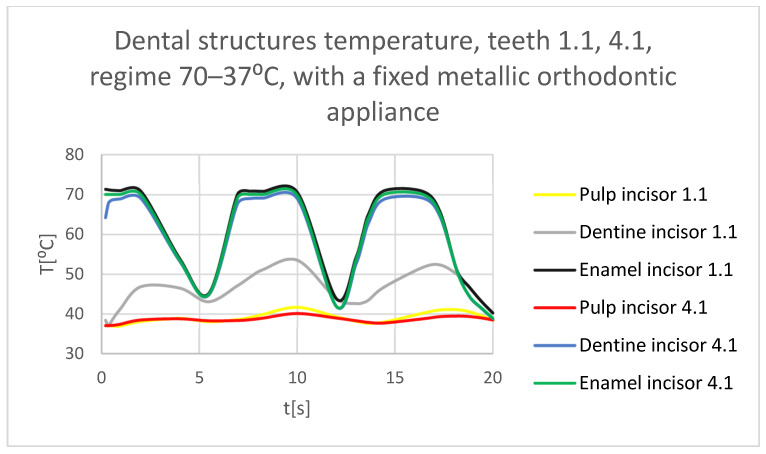
Comparative diagram of the dental structure temperature of teeth 1.1, 4.1 subjected to the hot thermal source.

Using the Probe-type virtual temperature sensors, the temperature diagrams of the dental enamel (Figure 46), of the dentine (Figure 47), and of the pulp (Figure 48) were obtained for tooth 1.1, the comparative diagram of the dental structures was obtained for tooth 1.1 (Figure 49), the temperature diagrams of the dental enamel (Figure 50), of the dentine (Figure 51), and of the pulp (Figure 52) were obtained for tooth 4.1, the comparative diagram of the dental structures was obtained for tooth 4.1 (Figure 53) and the comparative diagram of the dental structures was obtained for teeth 1.1 and 4.1 (Figure 54).

### 3.4. Thermal Simulation Results for the Stomatognathic System with a Fixed Metallic Orthodontic Appliance Having a Cold Temperature Source

**Figure 55:**
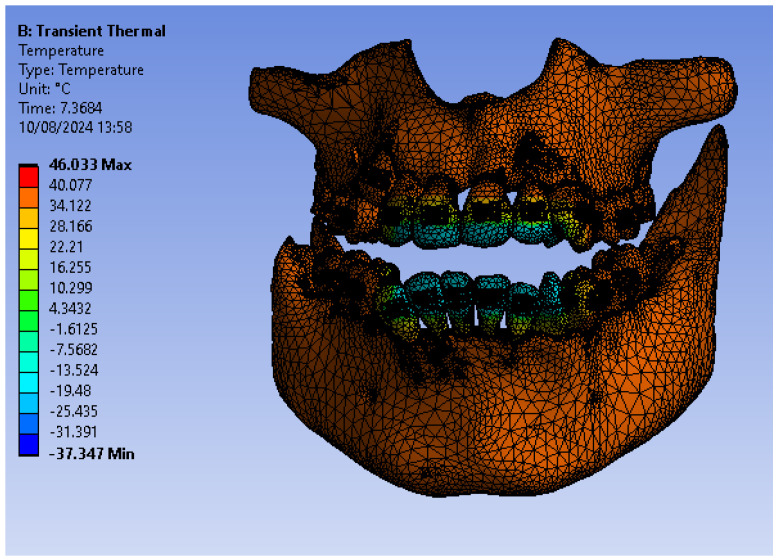
Temperature map.

Figure 55 shows the temperature map of the system subjected to thermal simulation.

**Figure 56:**
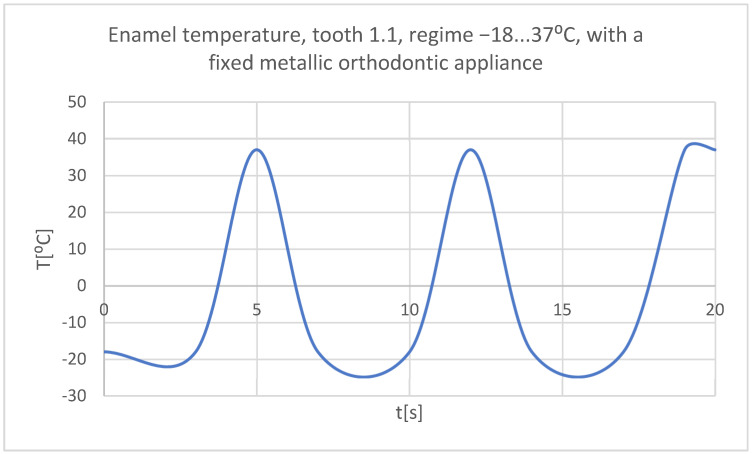
The temperature in the dental enamel of tooth 1.1 subjected to the cold thermal source.

**Figure 57:**
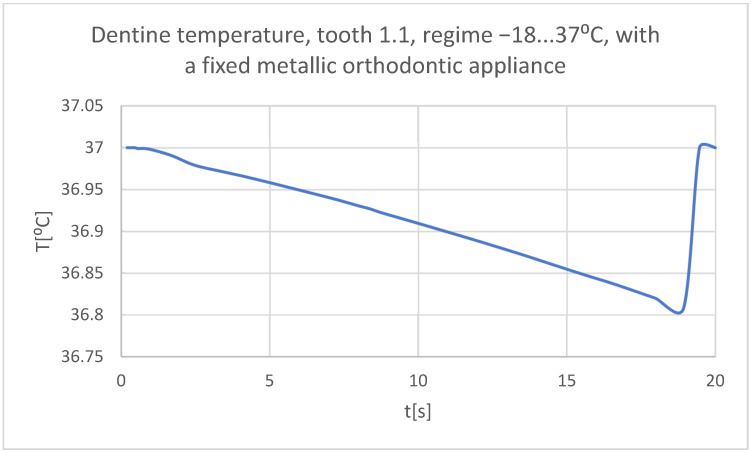
The temperature in the dentine of tooth 1.1 subjected to the cold thermal source.

**Figure 58:**
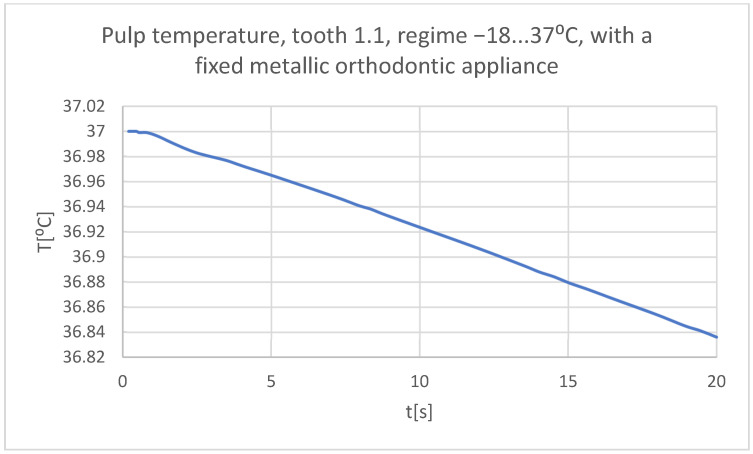
The temperature in the pulp of tooth 1.1 subjected to the cold thermal source.

**Figure 59:**
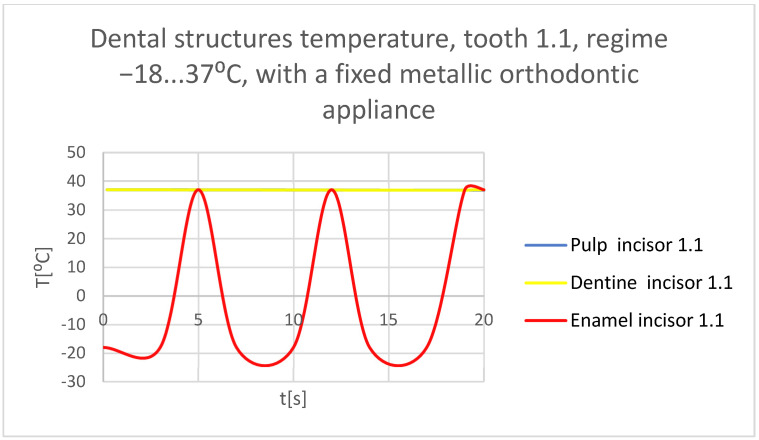
Comparative diagram of the dental structure temperature of tooth 1.1 subjected to the cold thermal source.

**Figure 60:**
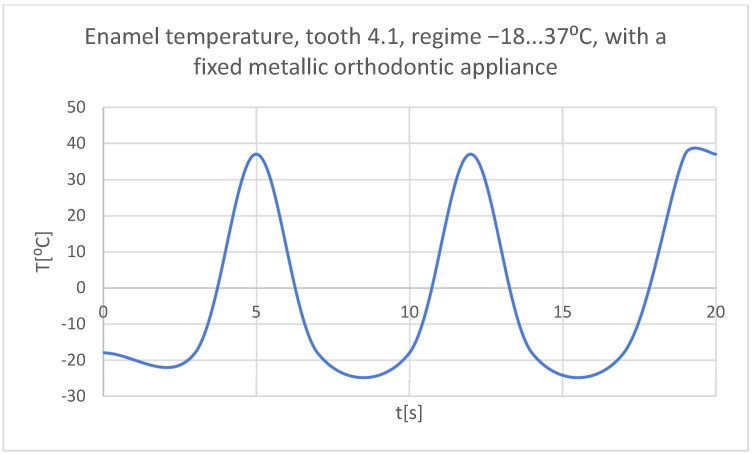
The temperature in the dental enamel of tooth 4.1 subjected to the cold thermal source.

**Figure 61:**
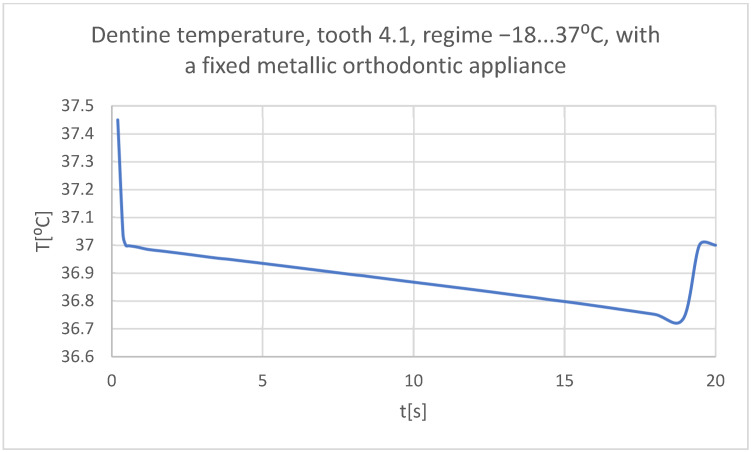
The temperature in the dentin of tooth 4.1 subjected to the cold thermal source.

**Figure 62:**
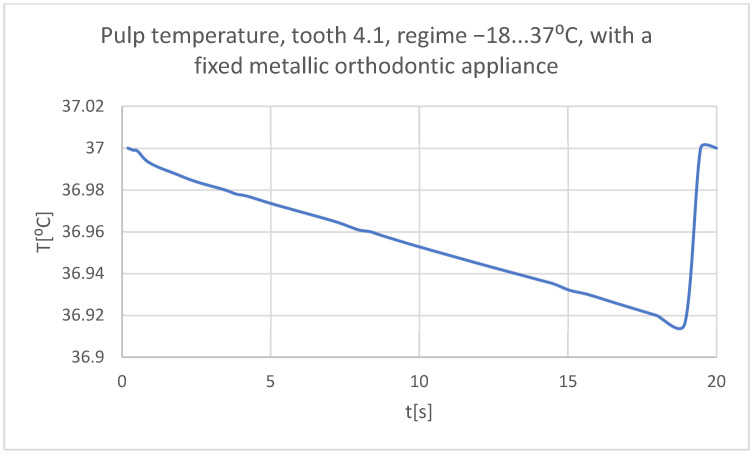
The temperature in the pulp of tooth 4.1 subjected to the cold thermal source.

**Figure 63:**
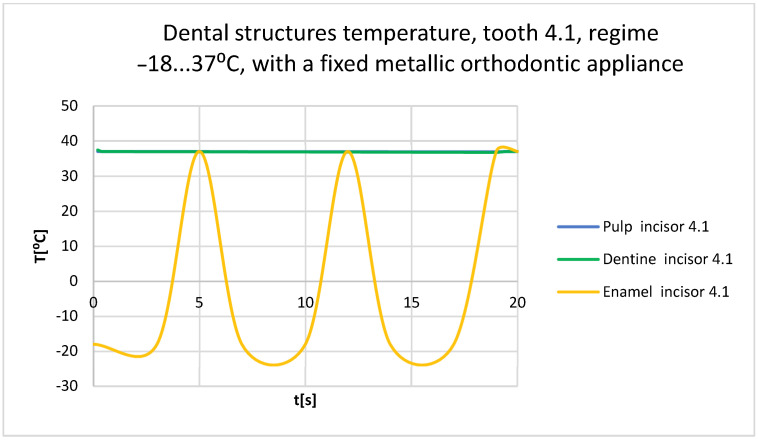
Comparative diagram of the dental structure temperature of tooth 4.1 subjected to the cold thermal source.

**Figure 64:**
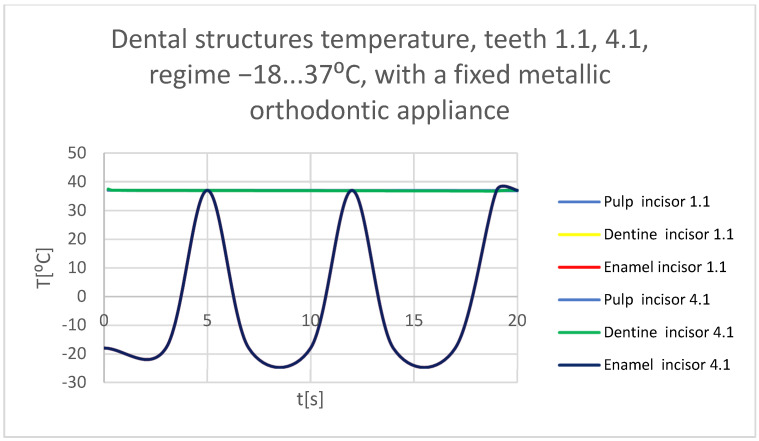
Comparative diagram of the dental structure temperature of teeth 1.1, 4.1 subjected to the cold thermal source.

Using the Probe-type virtual temperature sensors, the temperature diagrams of the tooth enamel (Figure 56), of the dentine (Figure 57), and of the pulp (Figure 58) were obtained for tooth 1.1, the comparative diagram of the dental structures was obtained for tooth 1.1 (Figure 59), the temperature diagrams of the dental enamel (Figure 60), of the dentine (Figure 61), and of the pulp (Figure 62) were obtained for tooth 4.1, the comparative diagram of the dental structures was obtained for tooth 4.1 (Figure 63), and the comparative diagram of the dental structures was obtained for teeth 1.1, 4.1 (Figure 64).

### 3.5. Comparative Diagrams

**Figure 65:**
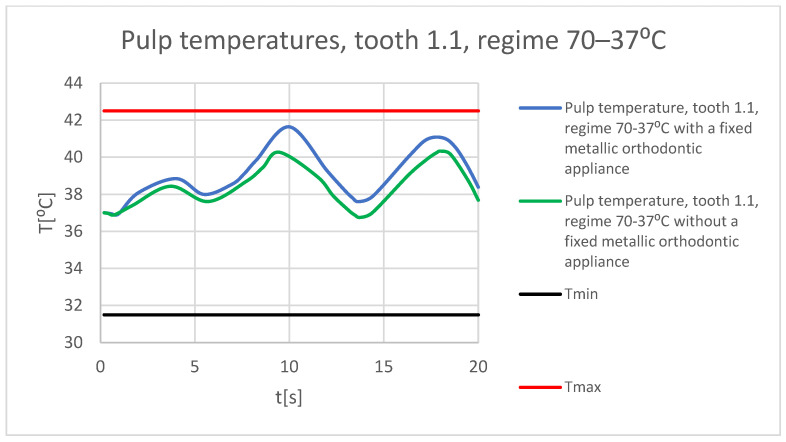
Comparative diagram of the temperature effect on the dental pulp (hot thermal source) for tooth 1.1.

It is known from the specialized literature that changing the temperature of the dental pulp by 5.5 °C plus or minus can produce irreversible damage [18]. This finding assumes that, in the range of 31.5 °C…42.5 °C, the dental pulp is intact and functional. In the following figures, Tmin = 31.5 °C and Tmax = 42.5 °C. Figure 65 shows a comparative diagram of the temperature effect on the dental pulp, with the hot thermal source, for tooth 1.1.

**Figure 66:**
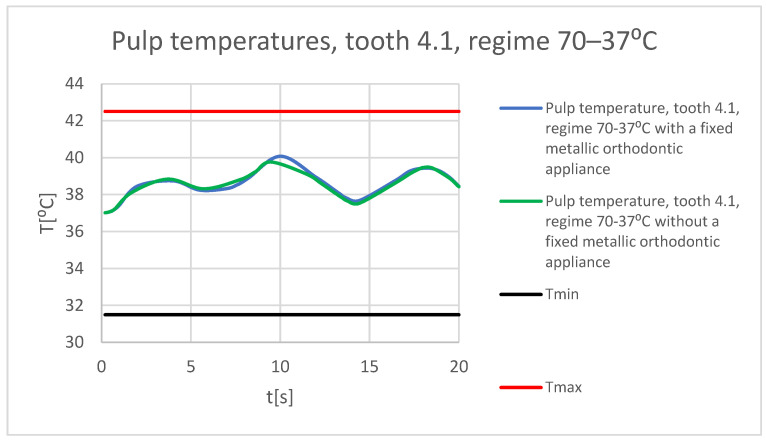
Comparative diagram of the temperature effect on the dental pulp (hot thermal source) for tooth 4.1.

Figure 66 shows a comparative diagram of the temperature effect on the dental pulp, with the hot thermal source, for tooth 4.1.

**Figure 67:**
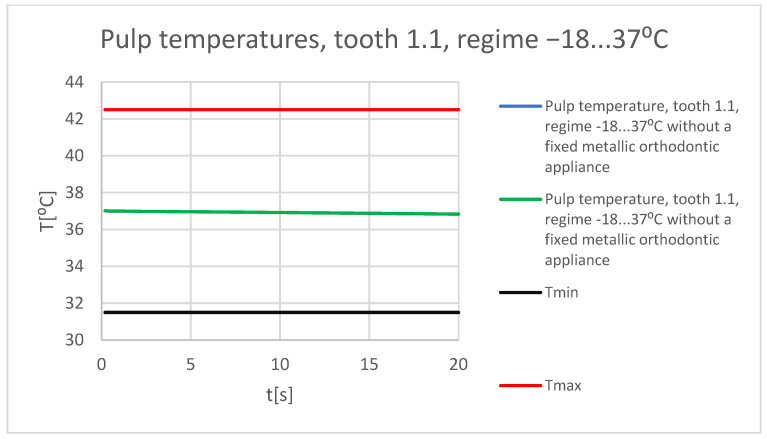
Comparative diagram of the temperature effect on the dental pulp (cold thermal source) for tooth 1.1.

Figure 67 shows a comparative diagram of the temperature effect on the dental pulp, with the cold thermal source, for tooth 1.1.

**Figure 68:**
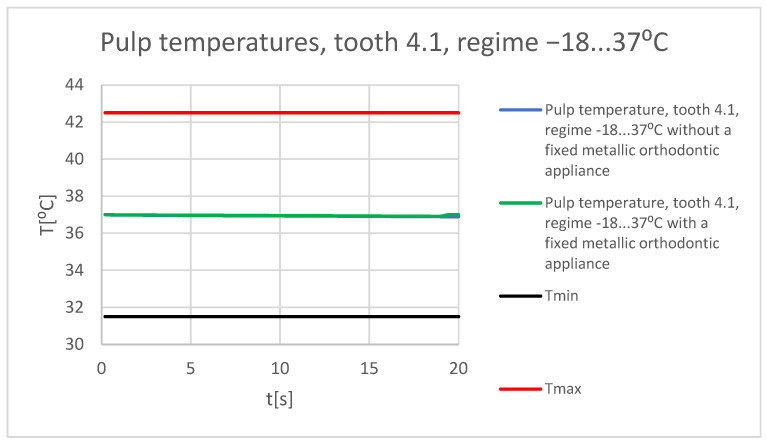
Comparative diagram of the temperature effect on the dental pulp (cold thermal source) for tooth 4.1.

Figure 68 shows a comparative diagram of the temperature effect on the dental pulp, with the cold thermal source, for tooth 4.1.

## 4. Discussion

In the specialized literature, there are few studies examining thermal influence on different stomatognathic apparatus structures using FEM.

In the early period of FEM application, many simulations did not directly lead to expected results, but indirectly, they generated important results in the field of materials testing or in the nonlinear analysis of force application in virtual human joints [19]. Initially, the analyzed models had a simplified geometry, and the division into finite elements was brief; currently, by improving the relevant methods, multi-body structures with complicated loading configurations and complex constraints can be analyzed. Also, a series of physical phenomena have been interconnected and can be studied simultaneously as simultaneous actions, such as thermal and structural study, and, through the emergence of complex subroutines, rigid and flexible components with linear or nonlinear mechanical behavior can be analyzed at the same time. In recent years, important progress has been made not only in the precise definition of the problem, but also in the multidisciplinary approach [20,21].

The FEM is used to replicate an orthodontic system in order to determine the elastic forces that arise when a patient is wearing a fixed metallic orthodontic appliance. This study used CBCT scans from a patient who presented a malocclusion treated with the help of a fixed metallic orthodontic appliance. Maxillary and mandibular orthodontic wires, a set of brackets, and tube-type elements were among the orthodontic components three-dimensionally processed. A model of the elastic forces that appear during fixed orthodontic therapy when a wire deforms was obtained using Ansys Workbench. As a result, the maximum deformation, maximum mechanical displacement, maximum stress, and deformation energy were determined [5].

The FEM can also be used to determine the odonto-periodontal stress of a real orthodontic system which has sustained various loads. To investigate this system, researchers used the case of a subject who was diagnosed with Angle class II malocclusion. With the help of the InVesalius program, DICOM-type images obtained from CBCT scans were transformed into three-dimensional structures. After editing, modifying, completing, and analyzing the three-dimensional structures with Geomagic software, the result was a three-dimensional model composed of perfectly closed surfaces, which can be converted into virtual solids. CAD programs were used to load this model.

Bracket and tube-type elements and also orthodontic wires were added to the model. The complete geometrical model was exported to the Ansys Workbench program, which uses the FEM. The researchers performed simulations for the forces from 0.5 to 1 N. Following these simulations, displacement, strain, and stress were displayed on result maps. After the study, the researchers discovered that periodontal ligaments and orthodontic wires provide elasticity to the orthodontic system, apart from its known rigidity [6].

With varying degrees of periodontal tissue injury, a study using the FEM assessed the maximal stress, orientation of force application, and displacement caused by the tooth–periodontal ligament–alveolar bone complex. The study showed that it is challenging to measure the forces produced while a patient is wearing a fixed orthodontic appliance from a clinical perspective. For affected teeth, the researchers advise that the intensity of these forces should not be higher than 1 N [22].

The biomechanical behavior of fixed orthodontic retainers was also highlighted with the help of the finite element method. Of particular interest was how their stiffness and tooth resilience had an impact on the transmission of force and on the distribution of stress. Using the FEM, the authors of the study obtained a virtual model of the lower maxillary from canine to canine with a retainer bonded on the lingual surface of the six teeth. The transmission of the force increased from 2 to 65% when axial or oblique stress was applied to the central incisors, as retainer stiffness and tooth resilience increased. Also, the researchers concluded that reducing the retainer diameter decreased the uniformity of the load disposition in the bonding interfaces. This causes the appearance of concentrated stress peaks inside a reduced part of the bonding area. Thus, they recommend avoiding using fixed orthodontic retainers with increased stiffness, particularly on high-resilience teeth [23].

The limitations of our study are represented by the fact that we did not take into account the adhesive used for the bonding of the fixed metallic orthodontic appliance, which is found in a very thin layer between the base of the bracket or tube-type elements and the surface of the enamel. At the moment, the adhesives used in the field of orthodontics have thermal conductivity close to that of enamel, which is why we considered it to not influence the results of our study.

In the future, we want to expand our research with similar FEM studies, alternating at short time intervals the thermal sources (cold–hot–cold and hot–cold–hot) to which various teeth are subjected.

## 5. Conclusions

The FEM is an accurate method that can be used to generate three-dimensional virtual models of the stomatognathic apparatus and to simulate the influence of different thermal stimuli on tooth structures.

This study’s findings indicate that any kind of FEM analysis can be performed beginning with a patient’s pre-treatment CBCT scans and virtual representations of the orthodontic wires, brackets, and tube-type elements.

From the analysis of the obtained data, it was concluded that, following the simulations carried out featuring the fixed metallic orthodontic appliance, significantly higher temperatures were generated in the dental pulp. Therefore, the quality of the materials used to manufacture orthodontic adhesives, metallic brackets, and tube-type elements can be increased with the help of FEM analyses.

## Figures and Tables

**Table 1:** The physical and thermal properties of the materials.

Component	Density [kg/m^3^]	Isotropic Thermal Conductivity [W · m/°C]	Specific Heat [J · kg/°C]
Enamel	2958	0.93	710
Dentine	2140	0.36	1302
Dental pulp	1000	0.0418	4200
Mandible, maxillary	2310	1	2650
Bracket and tube-type elements, Ni + Cr alloy	8500	13	460
Orthodontic wires, Ni + Ti alloy	6450	60	457

## Data Availability

The authors declare that the data from this research are available from the corresponding authors upon reasonable request.
